# Study on complications of osteoporosis based on network pharmacology

**DOI:** 10.3389/fgene.2022.941098

**Published:** 2022-09-29

**Authors:** Zhijing Song, Haoling Zhang, Yuhang Jiang, Rui Zhao, Xuedong Pei, Haochi Ning, Hailiang Chen, Jing Pan, Yanlong Gong, Min Song, Wei Wang

**Affiliations:** ^1^ Clinical College of Chinese Medicine, Gansu University of Chinese Medicine, Lanzhou, Gansu, China; ^2^ Affiliated Hospital of Gansu University of Chinese Medicine, Lanzhou, Gansu, China; ^3^ Key Laboratory of Dunhuang Medicine, Ministry of Education, Lanzhou, Gansu, China; ^4^ St Petersburg State University, St. Petersburg, Russia; ^5^ School of Public Health, Gansu University of Chinese Medicine, Lanzhou, Gansu, China; ^6^ Gansu University of Chinese Medicine College of Acupuncture-Moxibustion and Tuing, Lanzhou, Gansu, China

**Keywords:** network pharmacology, gene sequencing, guben zenggu granules, continuous static pressure, MC3T3-E1 cells

## Abstract

Osteoporosis is a serious threat to human life. Guben Zenggu Granule is an empirical prescription for clinical treatment of osteoporosis. MC3T3-E1 cells are mouse osteogenic precursor cells with osteogenic differentiation, and are classic cells for studying bone metabolism and osteogenic mechanism, as well as mechanical stimulation sensitive cells. Therefore, it can be inferred that Guben Zenggu granule can repair MC3T3-E1 cells under continuous static pressure overload. This study aims to through the network of pharmacology and gene sequencing method, reveal thrift increase bone particles under the condition of continuous static pressure overload on osteogenesis mechanism of MC3T3-E1 cells. In the process of analysis, from a variety of 98 compounds was predicted in the database, a collection of 474 goals, a total of 29,164 difference between two groups of genes. Then, construction of composite targets between cells and predict targets and protein - protein interaction networks, and through the cluster analysis to further explore the relationship between the target. In addition, linkages between target proteins and cells were further identified using Gene Ontology (GO) and Pathways (KEGG Pathway). Finally, the repair effect of Guben Zenggu granule on MC3T3-E1 cells under continuous static pressure overload was verified through experiments, so as to accurately explain the pharmacodynamic mechanism of Traditional Chinese medicine.

## Introduction

Osteoporosis (OP) is a common disease and frequently-occurring disorder of bone metabolism. The main clinical manifestations are bone loss and systemic chronic pain, and the main complications are brittle fracture (Gallagher et al., 1994; Gali, 2001; Zhu et al., 2021). In recent years, with the aging of the society, the disability rate caused by OP has increased significantly. The NUMBER of osteoporotic fractures is expected to rise to 4.5 million a year, according to a European Union study (Kanis et al., 2019). The number is expected to reach 18 million worldwide by 2040 (Yaacobi et al., 2017).

Guben Zenggu granule is professor Song Min’s experience prescription in clinical treatment of osteoporosis. It is mainly composed of astragalus membranaceus, codonopsis, angelica, epimedium, cistanche deserticola, rehmannia glutinosa, psoraleae, turtle worm, dog ridge, aconite, antler gum, and other drugs. The compatibility of Junchen Decoction with Traditional Chinese medicine has multiple targets and multiple effects in the treatment of osteoporosis, which is more scientific (Li et al., 2020; Ai et al., 2020). In the treatment of osteoporosis, TCM should focus more on compound studies, integrate the manifestations of TCM syndrome elements, summarize the characteristics of symptoms, give play to the compatibility advantages of compound therapy of king, minister and assistant and syndrome differentiation, and provide microcosmic material basis and support for the theory of “kidney main bone” (Zhao et al., 2021a; Cui et al., 2018; Zhang et al., 2016). Preliminary clinical studies have shown that Guben Zenggu granule can increase bone mineral density and effectively improve patients with osteoporosis pain, with definite clinical efficacy. Basic studies have also shown that Guben Zeng gu granule can promote the osteogenic differentiation of BMSCs and increase the content of BGP, OPN, ALP, and COLI proteins, possibly through the activation of BMPSmad/RUNX2 signaling pathway (Song, 2020a), (Song, 2020b). Guben Zenggu granule can reduce BGP and Trap-5B contents in serum and free [Ca^2+^] I concentration in bone in ovariectomized rats, thus regulating bone mineral density and stimulating biomechanical properties of bone tissue (Song, 2020c). Guben Zenggu granule and hyperbaric oxygen in the synergistic treatment of osteoporosis rats can effectively promote the balance between osteogenesis and osteofragmentation and enhance the activity of bone microstructure through the intervention and regulation of OPG/RANKL signaling pathway (Feng et al., 2021).

**FIGURE 1:**
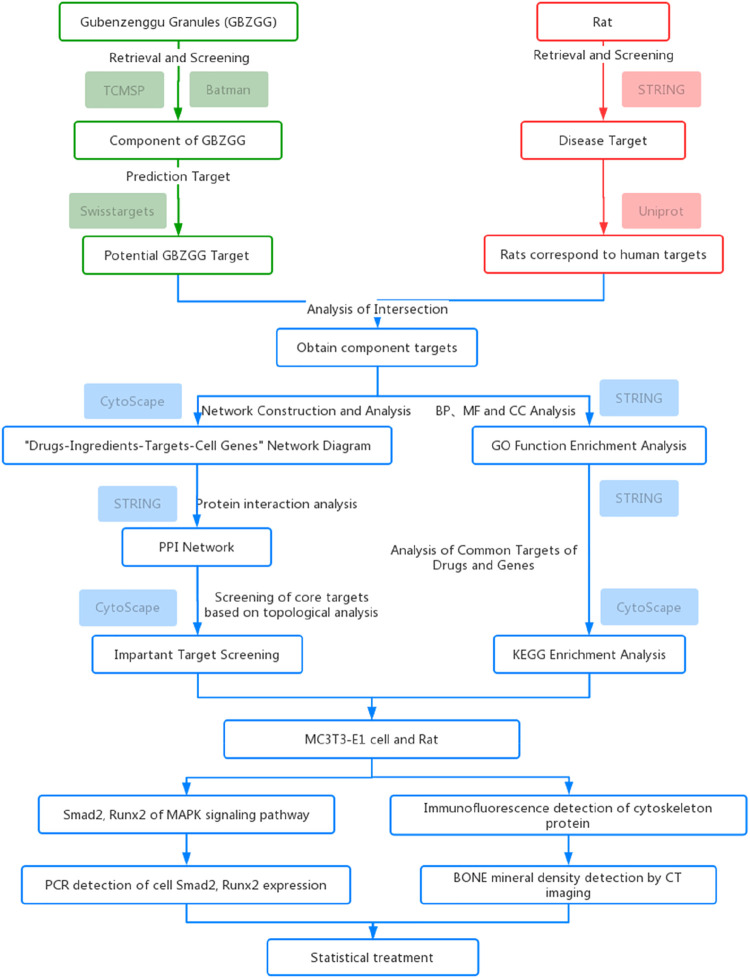
Shows the experimental design route in this paper.

Network pharmacology is a new method of pharmacological research (Li et al., 2017). It can identify and predict its related targets, bioactive compounds, and clarify the molecular mechanism of TCM. The core concept of TCM network pharmacology is “network target, multi-component” model, which can systematically elucidate the molecular mechanism of TCM treatment of various diseases (Xu et al., 2021; He et al., 2021). At present, there have been many pharmacological studies on network, such as gegen Qinlian Decoction for the treatment of type 2 diabetes, Wumei pill for the treatment of pancreatic tumor, and Baizhu root for the treatment of osteoporosis (Li et al., 2014; Wan et al., 2019; Zhang et al., 2019). Molecular docking technology is a new research method of computer-aided medicine, can through the computer to study the interaction between drug and target genes. Through the interaction between ligand and receptor, the binding pattern and affinity between ligand and receptor are predicted by computer data (Qiao, 2015). In this study, using computer-aided drug research method, the differentially expressed genes in MC3T3-E1 cells were obtained as the target of continuous static pressure overload intervention in MC3T3-E1 cells, and the effective components and pharmacological mechanism of Guben Zenggu granule in treating OP were studied. The experimental design route of this paper is shown in Figure 1.

## Experimental equipment

### Experimental cells

MC3T3-E1 cells were purchased from Wuhan Punosai Life Science and Technology Co., LTD. (Article number CL-0378).

#### Experimental software

TCMSP database (http://tcmspw.com/tcmsp.php) (Ru et al., 2014), Batman database (http://bionet.ncpsb.org/batman-tcm/) (Liu et al., 2016), the STRING database (https://string-db.org/) (Szklarczyk et al., 2019), Venny2.1 online software mapping tools (tools/venny/https://bioinfogp.cnb.csic.es/platform, David database (https://david.ncifcrf.gov/) Uniprot database (https://www.uniprot.org/) (Soudy et al., 2020), Cytoscape3.7.2 software, R3.6.1 software, etc.

### Main reagents and instruments

Micropipette (Eppendorf), Electrophoresis instrument power supply (Beijing Liu yi Instrument Factory), vertical electrophoresis tank (Beijing Liu yi Instrument Factory), electric rotary instrument (Beijing Liu Yi Instrument Factory), horizontal shaker (Jiangsu Haimen Qi Lin Bell Instrument Manufacturing Co., Ltd), pH meter (Metter-Toledo GmbH), Electronic balance (Beijing Sartoris Instrument System Co., Ltd.), magnetic stirrer (Zhongda Instrument Factory, Jintan city, Jiangsu Province), Enzyme labeler (Thermo) centrifuge (Hunan Xiangyi Laboratory Instrument Development Co., Ltd.) phosphatase inhibitor (Biyuntian), PMSF (Alding), RIPA lysis fluid (Biyuntian), BCA Protein Concentration Determination Kit, TEMED (Sinopharm Chemical Reagents Co., Ltd.), TrISE-Base (Biofroxx), HCl (Xinyang Chemical Reagents Co., Ltd.), DTT (Biofroxx), SDS (Sinopharm Chemical Reagents Co., Ltd.), Bromophenol blue (Sinopharm Chemical Reagents Co., Ltd), Glycerin (Sinopharm Chemical Reagents Co., Ltd), 30% acrylamide (Biosharp), TrisE-Base (Biofroxx), glycine (Biofroxx), SDS (Sinopharm Chemical Reagents Co., Ltd.), Tris-base (Biofroxx), *Glycine* (Biofroxx), Twain 20 (Sinopagic Chemical Reagents Co., Ltd.), Protein Marker (10-250kD), PVDF membrane (0.45 μm) (Millipore), PVDF membrane (0.22 μm) (Millipore), mouse monoclonal antibody β-actin (40KD) (Wuhan Bod Bioengineering Co., Ltd.), rabbit polyclonal antibody NOX4 (62KD) (Wuhan Sanying Biotechnology Co., Ltd.), mouse monoclonal antibody RANKL (35KD) (Abcam), Rabbit polyclonal antibody OPG (60KD) (Abcam), rabbit polyclonal antibody ColI (129KD) (Abcam), rabbit polyclonal antibody OC (11KD) (Wuhan Sanying Biotechnology Co., Ltd.), rabbit polyclonal antibody OPN (60KD) (Abcam), mouse monoclonal antibody Runx2 (57KD) (Abcam), HRP labeling sheep fight two resistance in mice boster biological engineering co., LTD. (wuhan), HRP labeling sheep rabbit 2 resisting boster biological engineering co., Ltd. (wuhan), ECL substrate liquid Pulitzer gene technology co., Ltd. (Beijing), X-ray film (c sharp cosette door medical equipment co., Ltd.), developing fixing kit (tianjin hanzhong photographic materials plant).

## Experimental methods

### Cell culture and passage

MC3T3-E1 cells (Wuhan Penosai Life Science and Technology Co., Ltd., Article NO. Cl-0378) were taken out of liquid nitrogen using MEM -α + 10%FBS + 1% (Penicillin Streptomycin Solution) cell medium, and quickly put into 37°C water bath. After dissolved, transfer the cells to contain 5 ml medium in the centrifuge tube. Centrifugation was performed at 1000 RPM for 5 min at room temperature, and the supernatant was discarded. In containing 10% fetal bovine serum (Gibco, No. 10099-141) the complete culture medium of cell suspension, and inoculated into a petri dish. The gently blown and mixed cells were cultured at 37°C, 5% CO2 saturation and humidity. When the cell density reached 80%, subculture, with 0.25% trypsin digestion, collect MC3T3-E1 cells after termination of digestive cells. With PBS washing cells twice, at 1500 rpm, 5 min; Complete culture medium was added, cells were blown, single cell suspension was prepared, and the culture was expanded at 37°C and 5% CO2 saturation humidity at the ratio of 1:3.

### RNA extraction and gene sequencing

1ul RNA is extracted by TRizol method and quantified by Nanodrop instrument. According to the quantitative results, 500 ng 1% agarose is used for electrophoresis detection, dscDNA is synthesized and the end is supplemented, 12.5µl A-tailing buffer is added. 12.5µl A tail buffer was added to 17.5µl DNA, and the mixture was fully mixed. After 30min at 37°C, the splice was added for PCR enrichment and Qubit was used to quantify the RNA library. Illumina HiSeq3000 was sequenced on a Start CBOT instrument, and the HiSeq 2500 was run on the machine for 11 days before the data was converted into FASTQ format.

### Expression of differential genes in MC3T3-E1 cells under continuous static stress

Using DESeq2 software for screening differentially expressed genes between groups, with different meet | log2FC | 1 or higher and Pvalue 0.05 or less scope of differentially expressed gene screening of the difference between the two groups. For the differentially expressed genes screened between sample groups, bidirectional hierarchical clustering of genes and samples was conducted and heat maps were used to display the clustering parameters (Distance metric: Pearson correlation; Linkage rule: Average Linkage). Mfuzz clustering method was used to classify the expression patterns into 10 groups for the sample size greater than or equal to 6.

### Drug composition and target screening

In TCMSP database (https://tcmspw.com/tcmsp.php) to retrieve the astragalus, codonopsis, angelica, epimedium, desertliving cistanche, rehmannia glutinosa, malaytea scurfpea fruit, ground beetle, dog ridge, radix linderae, antler glue ingredient, the composition of the filter is set to the OB 30% or higher, DL 0.18 or higher. For those not included in TCMSP, Batman database is used for retrieval, and Uniprot database is used for standardization and unification of target names. Will be gained by the composition by TCMSP database and Swisstargets database (http://www.swisstargetprediction.ch/) to obtain ingredients targets.

### TCM—Component-target-cell gene network construction and analysis

Use Cytoscape 3.7.2 software builds “pharmaceutical ingredients - target cell gene” Network diagram, using the Network Analyzer function to analyze the main effective ingredients of TCM compound. Network Analyzer is used to conduct topology analysis on the Network graph. The number of associations between components and targets is represented by degree values. The larger Degree value indicates that the component is more important.

### PPI network construction and core target analysis

Drug-induced disease will be the common target of the input STRING search the database, the protein type is set to “*Mus* musus”, minimum threshold is set to 0.4, the interaction between PPI network build proteins interacting with each other.

### Core target screening based on topology analysis

With degree, median centrality, mean shortest path length and total centrality as reference standards, genes with higher scores than average were selected as core targets by degree ranking, and bar charts of the first 30 targets were drawn using R3.6.1.

### Gene ontology enrichment analysis

The biological processes (BP), molecular functions (MF) and cellular components (CC) of GO are rich in common targets for drug cell genes and are referenced in the String database. Items with correction *p* < 0.05 were screened out. Using R 3.6.3 software installation and reference clusterProfiler, rich plot and GGplot2 package bar and bubble chart.

### KEGG enrichment analysis

Common targets of drug cell genes were analyzed by KEGG pathway enrichment, and the items with *p* < 0.05 were screened by String database. Using R 3.6.3, after installing and referencing the clusterProfiler package, draw the bar and bubble charts.

### Selection of the dominant dose group

Take the treated MC3T3-E1 cells in good growth condition, adjust the cell density to 5 × 104/ml with MEM-α medium, and connect them to a 96-well plate with 100 μl cell suspension per well. In the meantime, set a blank group at 37°C Cultivate overnight (add 100 μl sterile PBS to the holes around the cell wells); Treat the cells separately according to the following different groupings and cell treatment settings, each group has 3 multiple wells, cultured at 37°C for 24h; control group; MC3T3-E1 + blank serum 5%, 10%, 20%, MC3T3-E1 + low-dose serum 1%, 5%, 10%, 15%, 20%; MC3T3-E1 + medium-dose serum 1%, 5%, 10%, 15%, 20%; MC3T3-E1 + high-dose serum 1%, 5%, 10%, 15%, 20%, after cell treatment, add 10 μl MTT to each well and incubate at 37°C for 3h; aspirate the medium, add 150 μl DMSO and shake for 10min; Microplate reader detects the absorbance value OD 568.

### Western blot detection

PVDF membranes were immersed in TBST (sealing fluid) containing 5% skim milk powder and sealed with a mixer at room temperature for 2 h. A diluted with sealing fluid resistance, the PVDF membrane were soaked in a resistance to the fluid of the incubation, 4°C incubation for the night. The PVDF membrane was thoroughly rinsed by TBST 5 times, 5min/time. Put up to 3 membranes in a dish. In the process of flushing, and pay attention to whether film adhesion on board wall or membrane whether overlap. Corresponding HRP labeling 2 fight after diluted with TBST - 1:50,000, soaked the PVDF membrane in the fluid of the second antibody incubation, and incubated with the table 2 h at room temperature. Fully wash PVDF membrane with TBST 5 times, 5min/time. The enhancement solution in the ECL reagent was mixed with the stable peroxidase solution in a ratio of 1:1. The working droplets were added to the PVDF membrane and reacted for several minutes until the fluorescence band appeared. Then the excess substrate solution was absorbed with filter paper. Cover with plastic wrap, press X-ray film, put in developer solution for development, fixation solution, and rinse the film.

### PCR detection

Add 200μ L chloroform, mix thoroughly several times, and let stand at room temperature for 5 min. Centrifugation was performed at 12000rpm at 4°C for 15min, and the results were divided into three phases: upper (RNA), middle (protein) and lower (DNA). Transfer the upper water phase (about 400 μl) into another 1.5 ml EP tube, add 400 μl isopropyl alcohol, mix well, and let stand at room temperature for 10min. After centrifugation at 12000rpm at 4°C for 10min, white RNA precipitates could be seen at the bottom of the tube. Abandon the supernatant, add 1 ml without rnase 75% rnase-free 75% ethanol, vortex mixing, 4°C, 10,000 RPM, centrifugal 5 min. Repeat Step 6 once. The supernatant was discarded, the RNA was dried in air for precipitation for 5–10min, and the precipitation was dissolved in 20μlDEPC water. With microspectrophotometer dissolved RNA2 mu L OD260, OD280 and OD260/OD280 value, calculate the purity and concentration of RNA. According to OD260/OD280 ratio to estimate the quality of RNA, the ratio of between 1.8 ∼ 2.0 conform to the requirements of the experiments. The total RNA concentration (μg/μ L) = OD260 × 40×10-3. Save the total RNA in - 80°C refrigerator to spare.

Primer sequences used for gene detection are as follows:

### Immunofluorescence detection of cytoskeleton protein

The climbing cell slides in the culture plate were immersed in PBS, and the slides are fixed with 4% paraformaldehyde, and normal goat serum is dripped on the slides, and the slides are sealed at room temperature; incubate in a humid box at 37°C, and add fluorescence. DAPI was added dropwise to incubate in the dark, the specimens were stained nucleus; the slides were fixed with fixing solution containing anti-fluorescence quenching agent and observed under fluorescence microscope and images were collected.

### Bone mineral density detection by CT imaging

The tissues were soaked in 4% paraformaldehyde and then fixed for 24 h. Micro-ct was used to detect the morphological indicators of the tissues. Skyscan1276 Micro-CT Scaner software was used for scanning, and the parameters were set as follows: Voltage: 100 KV; current: 100 µA; scanning spatial resolution: 10 µm; resolution: 4032 × 2688 pixel; rotation Angle: 0.3°; exposure: 500 ms. After scanning, use Data Viewer software for calibration, and then use CT-AN software to select the area of interest. Finally, CT Vox software was used for 3D reconstruction and analysis.

### HE

Gradient alcohol is used to dehydrate the tissue. The tissue blocks must be transparent after dehydrated by alcohol. The transparent agent (xylene) can be mixed with dehydrating agent and paraffin wax at the same time. It replaces the dehydrating agent and the paraffin wax penetrates the tissue smoothly. The transparent tissue blocks were dipped in three cylinders of paraffin (60°C) successively. Embedding is to encase wax-impregnated tissue blocks in paraffin blocks. The temperature of embedding wax should be slightly higher than that of immersion wax to ensure that tissue blocks and embedded paraffin wax are completely integrated. The tissue slices were placed in a 40°C water bath after being sliced by a Leica pathological slicer. Dip anti-stripping slides into the water to scoop slice, slice was attached with the appropriate placement of the slide, at 60°C baking in the oven for 3 h. Paraffin section in xylene (20 min) ⅰ - xylene (20 min) ⅱ - xylene iii (min) 20-ⅰ anhydrous ethanol (5 min) - ethanol (5 min) ⅱ-95% alcohol (5 min) - 90% alcohol (5 min) - 80% alcohol (5 min) -70% alcohol (5min), soak in distilled water for 5min. Section into Mayer’s hematoxylin (clean staining background, no differentiation required) dye for 5min, wash and soak in tap water and return to blue. With 1% water soluble eosin staining section 5 min, 30 s is washed with tap water.

### Statistical treatment

SPSS22.0 software was used for variance analysis of relevant data, and *p* < 0.05 was statistically significant.

## Experimental results

### Differential gene analysis

**FIGURE 2:**
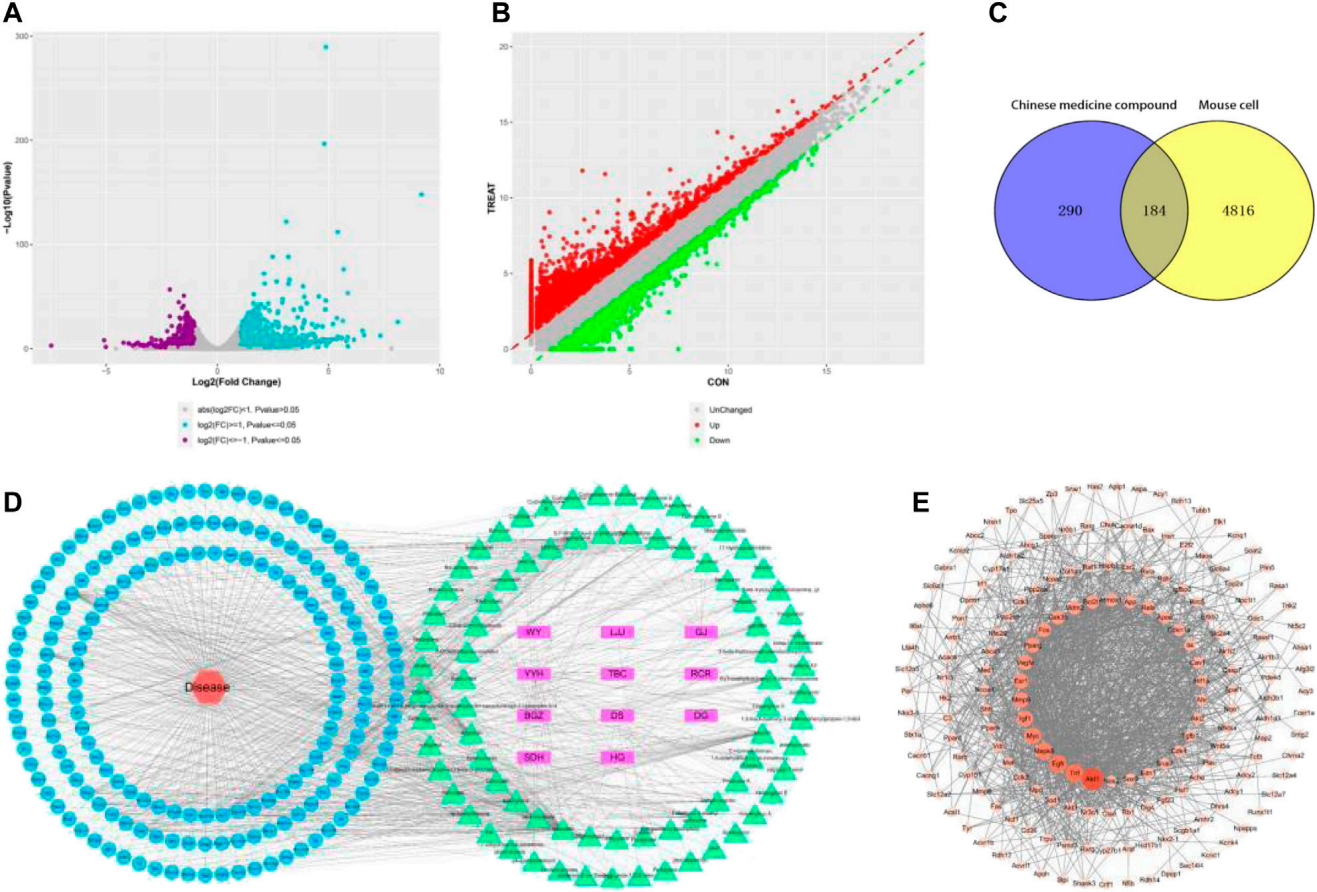
**(A)** Volcanic diagram between two groups of samples; **(B)** Scatter plot between two groups of samples; **(C)** Drug-cell gene common targets; **(D)** Drug-component-target-gene interaction network diagram; **(E)** Protein interaction network.

According to experimental design, using DESeq2 software is not the same screening differentially expressed genes between groups, according to | log2FC | 1 or higher and Pvalue 0.05 as differentially expressed or less range of filters, according to the results of 29,164 there were differences between two groups of genes, the expression level of 14,489, There were 14,675 down-regulated expressions, and Figures 2A,B were volcanic plots (Figure 2A) and scatter plots (Figure 2B) between the two groups of samples.

### Drug composition and target screening

OB ≥ 30% and DL ≥ 0.18 were set in THE TCMSP database to screen the active components and targets of Angelica sinensis, Codonopsis pilosula, *Astragalus* membranaceus, Cistanche deserticola, rehmannia glutinosa, Aconitum aconitum and Herba fularii. In the Batman database retrieval Dog‘s back, antler glue and tripelidae, received 98 potential active ingredients. Uniprot database was used to standardize and unify the target names and transform them into corresponding targets in mice. A total of 474 drug targets were screened out (Table 1).

**TABLE 1:** Drug composition of Guben Zenggu granule.

Seractial number	OB(%)	DL	herbs
MOL000358	36.91	0.75	DG、RCR、WY
MOL000449	43.83	0.76	DG、DS、SDH
MOL001006	42.98	0.76	DS
MOL002140	65.95	0.27	DS
MOL002879	43.59	0.39	DS
MOL003036	43.83	0.76	DS
MOL003896	42.56	0.20	DS
MOL004355	42.98	0.76	DS
MOL004492	38.72	0.58	DS
MOL005321	65.90	0.34	DS
MOL000006	36.16	0.25	DS、HYH
MOL006554	38.40	0.77	DS
MOL006774	37.42	0.75	DS
MOL007059	32.16	0.41	DS
MOL007514	39.67	0.23	DS
MOL008391	33.12	0.79	DS
MOL008393	38.33	0.29	DS
MOL008397	50.37	0.77	DS
MOL008400	50.48	0.24	DS
MOL008406	39.97	0.40	DS
MOL008407	45.40	0.76	DS
MOL008411	40.00	0.66	DS
MOL000211	55.38	0.78	HQ
MOL000239	50.83	0.29	HQ
MOL000296	36.91	0.75	HQ
MOL000033	36.23	0.78	HQ
MOL000354	49.60	0.31	HQ
MOL000371	53.74	0.48	HQ
MOL000374	41.72	0.69	HQ
MOL000378	74.69	0.30	HQ
MOL000379	36.74	0.92	HQ
MOL000380	64.26	0.42	HQ
MOL000387	31.10	0.67	HQ
MOL000392	69.67	0.21	HQ
MOL000398	109.99	0.30	HQ
MOL000417	47.75	0.24	HQ
MOL000422	41.88	0.24	HQ、HYH
MOL000433	68.96	0.71	HQ
MOL000438	67.67	0.26	HQ
MOL000439	49.28	0.62	HQ
MOL000442	39.05	0.48	HQ
MOL000098	46.43	0.28	HQ、RCR、WY、HYH
MOL005320	45.57	0.20	RCR
MOL005384	57.52	0.56	RCR
MOL007563	57.53	0.81	RCR
MOL008871	37.05	0.69	RCR
MOL000359	36.91	0.75	SDH、WY、HYH
MOL010495	31.93	0.30	WY
MOL010496	32.38	0.39	WY
MOL010907	40.92	0.46	WY
MOL010913	77.09	0.25	WY
MOL010916	42.55	0.19	WY
MOL010917	31.18	0.51	WY
MOL001510	37.58	0.71	HYH
MOL001645	42.10	0.20	HYH
MOL001771	36.91	0.75	HYH
MOL001792	32.76	0.18	HYH
MOL003044	35.85	0.27	HYH
MOL003542	38.04	0.39	HYH
MOL004367	62.23	0.41	HYH
MOL004373	45.41	0.44	HYH
MOL004380	39.14	0.49	HYH
MOL004382	56.96	0.77	HYH
MOL004384	45.67	0.50	HYH
MOL004386	51.63	0.55	HYH
MOL004388	60.64	0.66	HYH
MOL004391	48.54	0.25	HYH
MOL004394	41.58	0.61	HYH
MOL004396	52.31	0.22	HYH
MOL004425	41.58	0.61	HYH
MOL004427	31.91	0.86	HYH
MOL000622	63.71	0.19	HYH
The active ingredient			herbs
Corylifolinin			BGZ
Sophoracoumestan A			BGZ
Isopsoralidin			BGZ
Bavachin			BGZ
Bakuchiol			BGZ
Isobavachin			BGZ、GJ
Bavachalcone			BGZ
Bavachromene			BGZ
Psoralidin			BGZ
stigmasterol			BGZ
Xanthotoxin			BGZ
Backuchiol			BGZ
Angelicin			BGZ
Isobavachalcone			BGZ
Cudraphenone D			GJ
Kaempferol			GJ
Cudraphenone A			GJ
Bergapten			GJ
Naringenin			GJ
Aspidinol			GJ
Cudraphenone C			GJ
Cudraphenone B			GJ
Cudraflavanone B			GJ
Calcium Phosphate			LJJ
Calcium Carbonate			LJJ
Cholesterol			TBC

Note:DG:Angelica, RCR:Cistanche, WY:aconite, DS: Dangshen, SDH:cooked rehmannia glutinosa,RYH:epimedium,HQ:The root of remembranous milk vetch,BGZ:Psoraleae, GJ:dog spine,;LJJ:Antler glue,TBC:Ground beetle.

### Drug-cell gene common targets

Use Venny2.1 online software drawing tool platform, input 474 of the 5,000 drug targets and disease targets, map Venny2.1, for after the intersection of 184 drug cell gene common targets (Figure 2C).

### Traditional chinese medicine—composition—target—cell gene construction and analysis

98 out of 184 potential active components and drug-disease common targets in Traditional Chinese medicine compound were input into Cytoscape software, targets were deleted, overlapping components were separated, and the interaction network diagram of “drug component-target-gene” was drawn (Figure 2D). In the figure, purple represents drugs, green represents 80 active ingredients in TCM compound (18 active ingredient targets have no intersection with cell gene points and have been deleted, 80 ingredients have been marked red in Table 2), blue represents 184 common targets, and red represents cell genes.

**TABLE 2:** Primer sequence table.

Gene	Primer	Sequence (5′-3′)	PCR Products
b-actin	Forward	CAC​GAT​GGA​GGG​GCC​GGA​CTC​ATC	240bp
	Reverse	TAA​AGA​CCT​CTA​TGC​CAA​CAC​AGT	
*Mus* smad2	Forward	GAC​TAC​ACC​CAC​TCC​ATT​CC	233bp
	Reverse	GCA​GGT​TCC​GAG​TAA​GTA​A	
*Mus* Runx2	Forward	AGA​TGG​GAC​TGT​GGT​TAC​CG	203bp
	Reverse	TAG​CTC​TGT​GGT​AAG​TGG​CC	

### PPI network construction

Enter the above 184 common targets in the STRING database for a search. Protein type was set to “mice”, minimal interaction threshold value of 0.4. Get the target interaction network data, import Cytoscape software rendering protein interaction network diagram (Figure 2E). The Degree value is represented by the size, color, and shadow of the node.

### Gene ontology enrichment analysis

**FIGURE 3:**
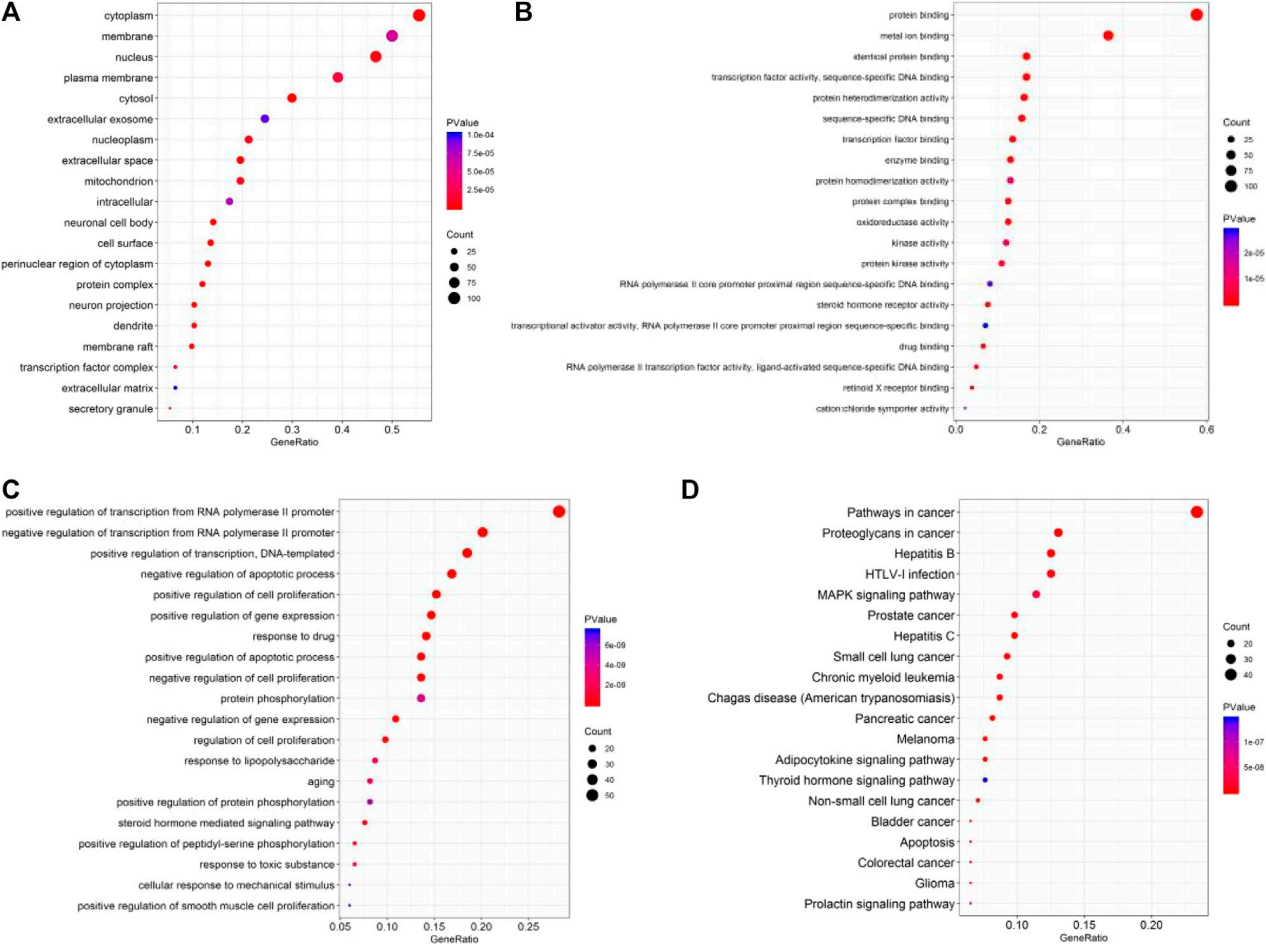
**(A)** GO Functional analysis (CC); **(B)** GO functional analysis (MF); **(C)** GO Functional analysis (BP); **(D)** KEGG analysis.

By GO enrichment analysis of David database, 184 common targets were obtained, and BP cross gene sets were enriched into 623 biological process pathways, mainly including positive regulation of RNA polymerase II promoter transcription, positive regulation of transcription and steroid hormone-mediated signaling pathways. CC intersection genes were concentrated in 71 cell components, mainly involving cytoplasm, cytoplasm, neuron cell body, protein complex, etc. In the process related to molecular function, the MF cross gene set was enriched to 140, mainly including protein binding, steroid receptor activity, protein heterodimerization activity, etc. See Figures 3A,B,C.

### KEGG enrichment analysis

By David database to enrichment of 184 common targets, received 107 KEGG pathways. Enrichment of top 20 results form the KEGG function bar chart (Figure 3D), including Pvalue representative enrichment of significance, the deeper the red color, the higher the significance. All the pathways shown here are Pvalue < 0.05, which is meaningful. You can choose the pathway you need based on the literature.

### Screening of the dominant dose group of drug-containing serum

After different concentrations of Guben Zenggu granule medicated serum on the proliferation of MC3T3-E1 cells, the results show that the concentration of medicated serum is too low, the promotion effect is not good, and the concentration is too high, its proliferation will be inhibited. The final 10% high-concentration medicated serum promoted the proliferation of MC3T3-E1 cells best, and its proliferation rate reached 116% (Table3).

**TABLE 3:** The effect of different concentrations of Gubenzenggu granule medicated serum on the proliferation of MC3T3-E1 cells.

Group	OD	Proliferation rate (%)
blank	0.0777 ± 0.0022	
The control group	1.0259 ± 0.0082	100 ± 0.01
5% Blank serum	1.0041 ± 0.0048	98 ± 0.02
10% Blank serum	1.0873 ± 0.0169	106 ± 0.01
20%Blank serum	0.9499 ± 0.0123	92 ± 0.01
1% Low concentration of drug-containing serum	1.0118 ± 0.0055	99 ± 0.04
5% Low concentration of drug-containing serum	1.0495 ± 0.0342	102 ± 0.02
10% Low concentration of drug-containing serum	1.1247 ± 0.0162	110 ± 0.03
15% Low concentration of drug-containing serum	1.0179 ± 0.0270	99 ± 0.01
20% Low concentration of drug-containing serum	0.8874 ± 0.0141	85 ± 0.01
1% Medium concentration drug containing serum	1.0357 ± 0.0123	101 ± 0.04
5% Medium concentration drug containing serum	1.0529 ± 0.0364	103 ± 0.03
10% Medium concentration drug containing serum	1.1456 ± 0.0288	113 ± 0.02
15% Medium concentration drug containing serum	1.0394 ± 0.0157	101 ± 0.02
20% Medium concentration drug containing serum	0.8950 ± 0.0147	86 ± 0.01
1% High concentration of drug serum	1.0115 ± 0.0071	98 ± 0.02
5% High concentration of drug serum	1.0554 ± 0.0155	103 ± 0.03
10% High concentration of drug serum	1.1748 ± 0.0279	116 ± 0.01
15% High concentration of drug serum	0.9971 ± 0.0097	97 ± 0.02
20% High concentration of drug serum	0.8651 ± 0.0214	83 ± 0.01

### WB detection

**FIGURE 4:**
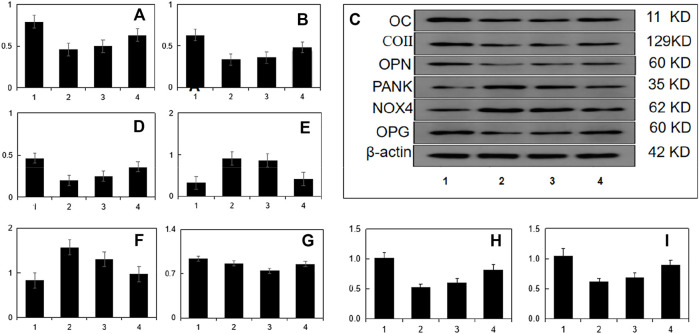
Continuous static pressure damage model index WB detection. Note:1:Blank control group 2:Model group3:Blank serum group 4:Drug containing serum group.**(A)**:OC/β-actin,**(B)**:CoⅡ/β-actin,**(D)**:OPN/β-actin,**(E)**:Rankl/β-actin,**(F)**:Nox4/β-actin,**(G)**:OPG/β-actin;**(H)**:RT-QPCR was used to detect TGF-β/BMP signaling pathway Smad2 in MC3T3-E1 cells. **(I)**Rt-qpcr was used to detect runx2 gene expression of TGF-β/BMP signaling pathway in MC3T3-E1 cells.

The results showed that the ratio of OC/β-actin, ColⅠ/β-actin, OPN/β-actin, OPG/β-actin in the blank serum group was higher than that in the stress model group (*p* < 0.05), while the drug-containing serum group was compared with its ratio increased (*p* < 0.05). The ratios of Rankl/β-actin and Nox4/β-actin in the drug-containing serum group were significantly reduced (*p* < 0.05). Therefore, it can be seen from the table that the intervention effect of the drug-containing serum group on sustained static pressure injury cells is more significant than that of the blank serum group. For details, see Table 4; Figure 4.

**TABLE 4:** Continuous static pressure damage model index WB detection (*n* = 3).

	Blank control group	Model group	Blank serum group	Drug containing serum group
OC/β-actin	0.793 ± 0.023	0.459 ± 0.053	0.498 ± 0.053	0.634 ± 0.082*
ColⅠ/β-actin	0.633 ± 0.063	0.337 ± 0.050	0.359 ± 0.042	0.484 ± 0.020*
OPN/β-actin	0.466 ± 0.027	0.199 ± 0.043	0.247 ± 0.043	0.361 ± 0.052*
Rankl/β-actin	0.316 ± 0.033	0.915 ± 0.021	0.869 ± 0.040	0.413 ± 0.043*
Nox4/β-actin	0.829 ± 0.045	1.569 ± 0.069	1.309 ± 0.042	0.974 ± 0.061*
OPG/β-actin	0.937 ± 0.063	0.863 ± 0.25	0.746 ± 0.046	0.850 ± 0.050

*Represents *p* < 0.05.

### Guben Zenggu Granule drug-containing serum can inhibit the expression of Smad2 and Runx2/Cbfa1 genes in MC3T3-E1 cells under continuous static pressure overload

To analyze the influence of Smad2 and RUNx2/Cbfa1 gene expression in MC3T3-E1 cells under continuous static pressure, the cells were grouped to verify the expression levels of Smad2 and Runx2. Results show that the cell model (0.5 mpa) of Smad2 and RUNx2 expression level is lower than normal group (*p* < 0.05). As shown in Figure 11-12. The above results showed that Smad2 and Runx2/Cbfa1 gene expressions were inhibited in MC3T3-E1 cells under continuous static pressure, while the expression of Smad2 and Runx2/Cbfa1 gene was up-regulated by guben Zenggu granule containing serum, and the expression was normalized (Figures 4H,I).

### Bone mineral density and bone trabecular examination

**FIGURE 5:**
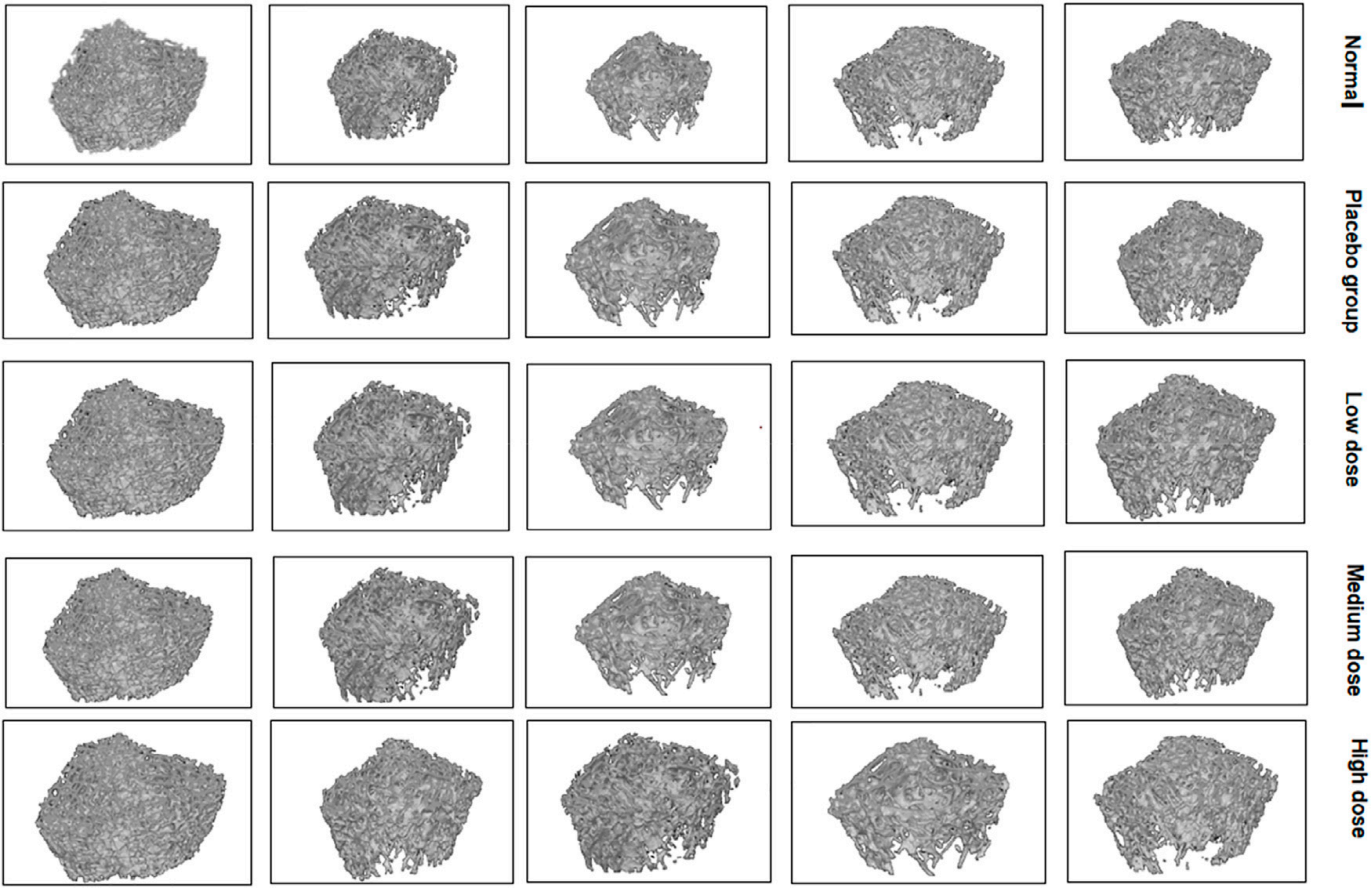
Image of trabecular bone.

**FIGURE 6:**
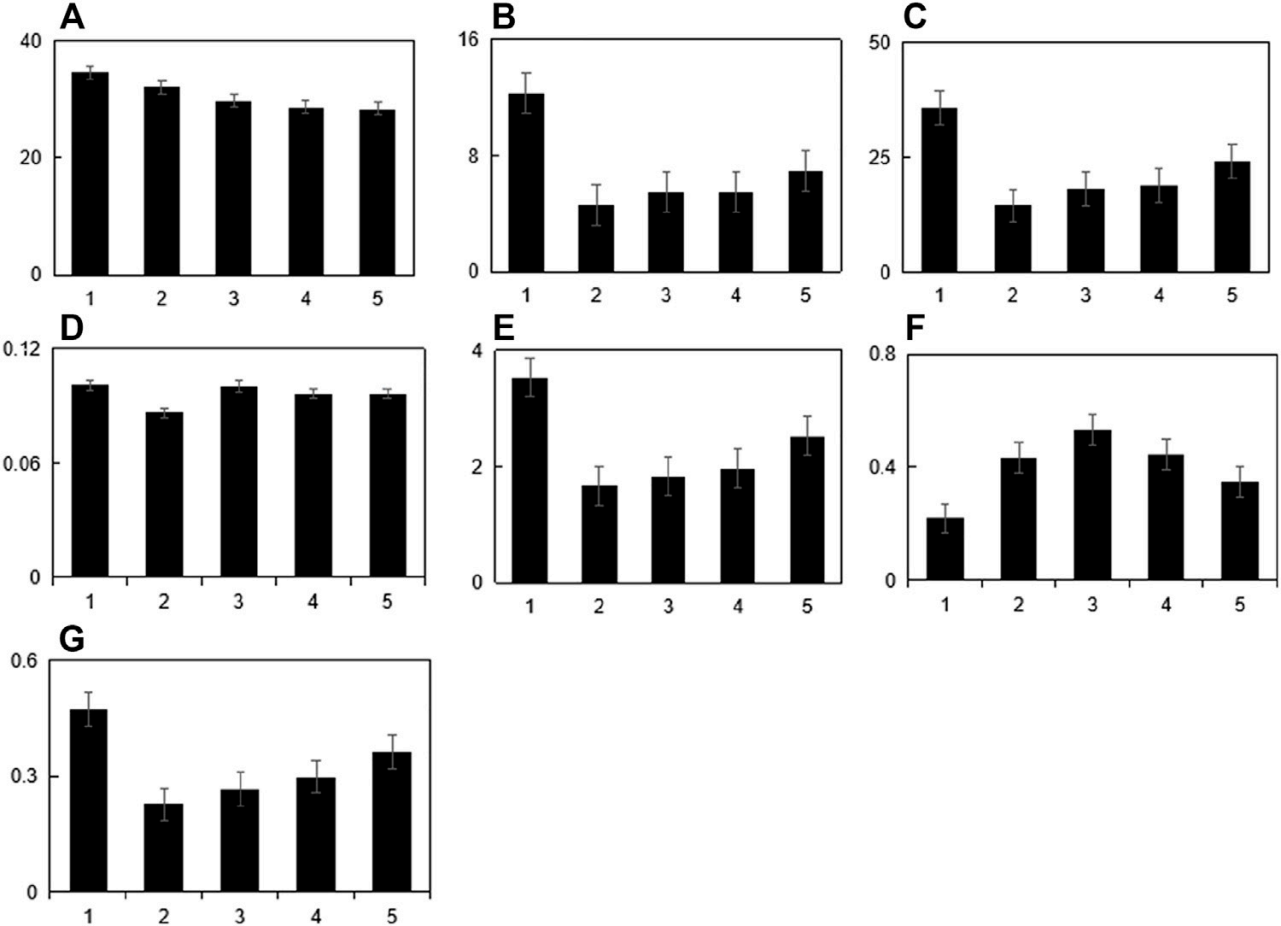
Bone mineral density index and bone trabecular index. Note: **(A)**TV MM3 (selected volume of ROI),**(B)**:BV MM3 (bone volume),**(C)**:BV/TV % (bone volume fraction),**(D)**:Tb.Th mm (bone trabecular thickness),**(E)**:Tb.N 1/mm (bone trabecular number),**(F)**:Tb (Bone trabecular separation), **(G)**BMD G/cm3 (bone density),1. Normal rats group,2. Rat model + placebo group, 3. Rat model + low dose,4. Rat model + medium dose5. Rat model + high dose* *represents p < 0.05.

As shown in Figures 5, 6, with the increase of dose, bone mineral density, bone, bone volume fraction volume, trabecular thickness, trabecular number and trabecular spacing returns to normal.

### HE dyed

**FIGURE 7:**
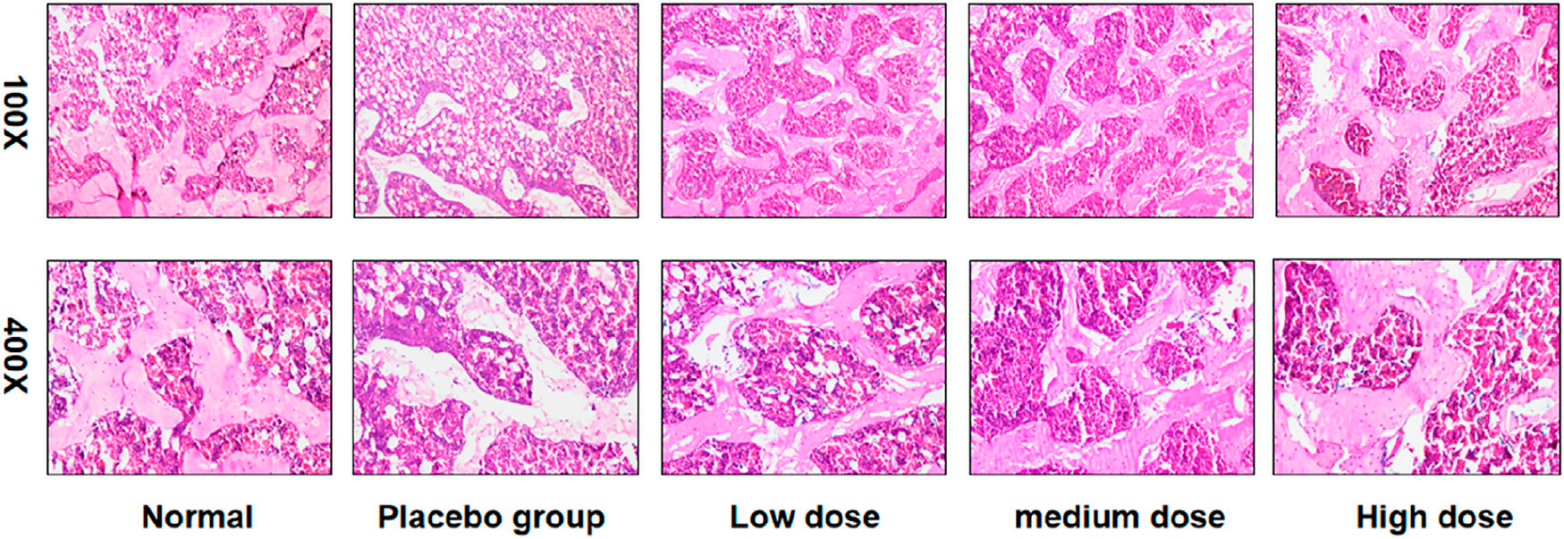
HE staining of bone tissue with different doses of drugs.

The high dose group had a significant effect on the repair of bone tissue injury compared with the low dose group, indicating that the drug had a repair effect. (Figure 7).

## Discussion

The doctor of traditional Chinese medicine in the treatment of the disease has a unique advantage. It has multi-targets and multi-effects in the treatment of diseases. The principle of compatibility of monarchs and ministers makes its application more scientific. However, the dosage is also one of the factors that affect its effect in the process of using Chinese herbal compound. Through the influence on the proliferation rate of MC3T3-E1 cells, the optimal concentration and dose in the drug-containing serum of different concentrations and doses are screened. As the concentration increases, the effect of promoting proliferation is stronger. However, high doses of different doses of medicated serum will inhibit the proliferation of MC3T3-E1 cells. Finally, in a concentration gradient, a 10% dose of medicated serum has the best effect on cell proliferation.

Osteoporosis is a common systemic bone disease, mainly due to the imbalance of bone metabolism, resulting in bone mass reduction and bone microstructure destruction. This study is based on the effective clinical basis of TCM and is the core approach to realize the modernization of TCM. Traditional Chinese medicine (TCM) is a kind of composition, ways and targets of natural medicine. In this study, cell sequencing genes were analyzed to clarify the basis and mechanism of drug action, and experimental verification was carried out.

In this study, KEGG and GO results showed that Guben Zenggu granule were involved in major signaling pathways including MAPK and positive regulation of cell apoptosis on MC3T3-E1 under continuous static pressure. The negative regulation of cell apoptosis process; Negative regulation of cell proliferation; Positive regulating cell proliferation; Negative regulation of gene expression; Regulate cell proliferation pathways.

MAPK signaling pathway, the bone morphogenetic protein (BMPs) signaling pathway is involved in a variety of bone metabolic processes through two typical Smad protein-dependent pathways (TGF-β/BMP ligand, Receptors and Smad proteins) and atypical Smad independent signaling pathways (MAPK signaling TGF-β/BMPs P38 mitogen-activated protein kinase signaling pathway). The former is indispensable in cell stress transduction and osteogenesis, mainly reflected in Smad’s regulation of TGF-β/BMP signaling pathway, while the latter directly affects cytoskeleton depolymerization and rearrangement, mainly reflected in Smad’s regulation of MAPK transduction signal. Runx2 is a downstream target gene of the TGF-β/BMP pathway. Runx2 and Smads activated by bone morphogenetic protein jointly induce osteoblast specific gene expression and regulate bone metabolism. The target of bone morphogenetic protein signal is Runx, and BMPs signal transduction pathway is involved in the physiological response of osteoblasts to stress stimulation, and is very important in the level of information transmission in this process. The “endpoint” of mechanical stress stimulation in osteoblasts was the up-regulation of Runx2/Cbfa1 gene expression.

Continuous static pressure is a typical physical factor in the study of osteoporosis. It has been shown in the clinic that an appropriate amount of pressure can promote the therapeutic effect of OP. The reason is that cells will produce a series of physiological and biochemical reactions after stress stimulation to resist the next pressure. Stimulate. In the related studies of continuous static pressure on MC3T3-E1 cells, OC, ColⅠ, OPN, RANKL, NOX4, OPG, osteocalcin, ADAM28, TD, ALP, Runx2, Wnt1, DKK-1, and other genes and their Protein expression is the main research direction (Song, 2021; Pengjam, 2021; You et al., 2001; Zhao et al., 2021b).

The osteoblasts responsible for bone formation activities are differentiated from bone marrow mesenchymal stem cells. Through exploring osteopontin (OPN), osteocalcin (OC), type II collagen (Col I), and osteoprotegerin (OPG) genes The expression of protein explores the molecular biological mechanism of cellular osteogenics. The results show that Guben Zenggu Granules medicated serum can regulate the osteogenic markers of MC3T3-E1, indirectly promote the osteogenic differentiation of BMSCs, and affect other bone formation indicators of osteoblasts. It has a promoting effect and can effectively improve molecular biology and anti-oxidation. The results are correlated with previous studies (Feng et al., 2021; Dong et al., 2019a; Dong et al., 2018; Dong et al., 2019b; Wei et al., 2019), confirming that Guben Zenggu Granules medicated serum has a significant effect on osteoporosis, and effectively demonstrates the advantages of traditional Chinese medicine in the treatment of diseases. Advantages of Chinese herbal compound treatment.

The positive regulatory signaling pathway of apoptosis is an important programmed cell death for metazoan development and internal environment stability. Apoptosis signaling pathway plays an important role in osteoclast - induced bone loss. Jijie Chai suggested that Smac/DIABLO could not only promote protein hydrolysis activation of procaspase-3, but also promote the enzyme activity of mature caspase-3 (Chai et al., 2000). As a targeted anti-apoptotic drug, Guben Zenggu granule may positively regulate the apoptosis process through MAPK. Negative regulation of apoptosis process; Negative regulation of cell proliferation; Positive regulating cell proliferation; Negative regulation of gene expression; Regulating cell proliferation pathway and Runx2, Smad gene protein acts on MC3T3-E1 cells to treat induction of bone resorption related diseases such as osteoporosis and fracture.

## The conclusion

As a targeted anti-apoptotic drug, Guben Zenggu granule may positively regulate the apoptosis process through MAPK. Negative regulation of apoptosis process; Negative regulation of cell proliferation; Positive regulating cell proliferation; Negative regulation of gene expression; Regulating cell proliferation pathway and Runx2, Smad gene protein acts on MC3T3-E1 cells to treat induction of bone resorption related diseases such as osteoporosis and fracture.

Too high a dose of medicated serum will inhibit the proliferation of MC3T3-E1 cells. In the final concentration gradient, a 10% dose of medicated serum has the best effect on cell proliferation. Guben Zenggu Granules medicated serum may directly participate in inducing the generation and differentiation of OB and OC by regulating TGF-β/BMP and BMP-2/Smads signaling pathways, and regulate bone metabolism in a continuous static pressure overload environment. This may be one of the effects of Guben Zenggu Granules medicated serum through regulating bone metabolism.

Guben Zenggu Granules medicated serum can promote the expression of Tubulin and actin proteins in MC3T3-E1 cells in a continuous static pressure overload environment, thereby promoting bone metabolism.

## Data Availability

The original contributions presented in the study are included in the article/supplementary material, further inquiries can be directed to the corresponding authors.
